# Heliconiini butterflies display flight behaviours reminiscent of orientation flights when using new floral sources

**DOI:** 10.1242/jeb.250975

**Published:** 2025-11-05

**Authors:** Denise Dalbosco Dell'Aglio, W. Owen McMillan, Stephen Montgomery

**Affiliations:** ^1^ Smithsonian Tropical Research Institute, Panama City 0843-03092, Panama; ^2^ School of Biological Science, University of Bristol, Bristol BS8 1TQ, UK

**Keywords:** *Heliconius*, Mushroom bodies, Foraging, Spatial learning, Spatial memory

## Abstract

Despite their small brains, many insects form long-term memories of the spatial distribution of resources. To support this, some species display ‘orientation’ flights to increase the capture of landscape cues around novel resources. The role of orientation behaviour in spatial learning has been broadly explored in Hymenoptera, which are well known to navigate between their nests and floral resources. Here, we describe exaggerated flight behaviours expressed in a foraging context in Heliconiini butterflies, resembling orientation behaviours in Hymenoptera. Heliconiini butterflies are of particular interest as they include the genus *Heliconius*, which is reported to have a greater capacity for spatial memory in association with a novel dietary strategy of pollen feeding, including formation of stable foraging routes. We hypothesised that these flight behaviours may provide a strategy to memorise the location of floral resources. We compared *Heliconius* and non-*Heliconius* butterflies to evaluate this ability in pollen- and non-pollen-feeding Heliconiini species. We characterized three behavioural patterns that are directed towards a new floral resource: circle flights, hovering and feeding bouts. Although all Heliconiini studied displayed these patterns, *Heliconius* spent more time performing these flight patterns, in particular hovering before landing. We suggest that these behaviours could provide the context in which individuals acquired sensory information to guide future foraging events, and our data indicate greater emphasis of this process during memory formation. Our findings of behaviours reminiscent of orientation flights in Heliconiini butterflies provide new avenues of research on how spatial learning and memory have converged between Lepidoptera and Hymenoptera.

## Teaser

**Summary:** Exaggerated flight behaviours towards new floral resources in Heliconiini butterflies may have a similar function to ‘orientation’ flights in Hymenoptera, as a strategy to memorise the location of floral resources.

## INTRODUCTION

Despite their small brains, insects exhibit incredible orientation behaviours that extend from short range flights for homing and food finding, to long-range behaviours such as long-distance migration and compass orientation (Beetz and el Jundi, 2023; Zeil, 2012). During foraging, associating environmental cues with reliable resources by repeated visitations allows for more efficient exploitation. Insect spatial behaviour has been explained by three mechanisms through which spatial memory can be acquired: alignment image-matching, positional image-matching and path integration (Collett et al., 2013). The first two are based on the wide-field landscape views of an individual's surroundings, a representation of which is formed in the brain, providing so-called ‘snapshot memories’, where informative landscape features are extracted from the retinal image (Cartwright and Collett, 1983). To reach a functional degree of accuracy, some insects extract information from the wide-field landscape around their resource during ‘orientation’ or ‘learning flights’ (Bates, 1863; Lehrer, 1993; Zeil et al., 1996), or in flightless species, ‘orientation walks’ (Collett, 2010). During these orientation behaviours, insects are thought to store representations of the landscape, creating a visual memory of that scene that allows them to return to the location of a rewarding resource (Capaldi and Dyer, 1999; Wystrach and Graham, 2012).

A role for orientation flights in learning landscape information has mostly been observed in foraging bees and wasps, which are well known to visually navigate when traveling between their nests and resource locations (Collett and Hempel de Ibarra, 2023). The structure of orientation flights is very specific, and similarities in certain details are found across different hymenopteran species (Zeil et al., 1996). The principles of these learning flights are that after leaving a foraging site, insects turn back several times to face the goal as they move away from it (Lehrer, 1993). They move in circles or loops, first reversing their direction, before pivoting around and returning to the goal until it has crossed the frontal visual field (Lehrer and Collett, 1994; Stürzl et al., 2016; Zeil et al., 1996). A second notable feature is that they decrease in size and frequency with increased familiarity of a resource location (Capaldi and Dyer, 1999; Capaldi et al., 2000; Menzel et al., 2006; Müller and Wehner, 2010). The functional significance of orientation behaviours is to allow individuals to learn certain aspects of the landscape as viewed from a fixed location, and then when they are returning to that location, they seek to match their representation of the learnt landscape features to their current visual field (Zeil, 2012). Indeed, in ants, the first learning walk of foragers is sufficient to alter synaptic complexes in the mushroom bodies (Ardin et al., 2016; Heinze, 2020; Stieb et al., 2010), the primary learning and memory centre in the insect brain (Farris, 2008; Zars, 2000), and retrieval of memories of foraging experience are associated with Kenyon cell activity (Buehlmann et al., 2020; Kamhi et al., 2020; Mizunami et al., 1998). Similarly, in honeybees, mushroom bodies show elevated levels of activity during foraging experience, and the number of active mushroom body neurons is related to the visual input received during the foraging flight (Kiya and Kubo, 2011).

Currently, orientation flights have been described exclusively in Hymenoptera, such as honeybees (Capaldi and Dyer, 1999; Capaldi et al., 2000; Cartwright and Collett, 1983), bumblebees (Riabinina et al., 2014; Robert et al., 2018), solitary bees (Wcislo, 1992) and wasps (Stürzl et al., 2016; Zeil et al., 1996), and some ants perform similar orientation walks (Collett, 2010; Collett et al., 1992; Müller and Wehner, 2010). However, like many flying Hymenoptera, butterflies exploit ephemeral floral resources that are scattered in space and vary in quality. The tropical butterfly genus *Heliconius* presents a particularly highly derived strategy in this context, uniquely exploiting pollen as a source of amino acids in adulthood, which supports a prolonged reproductive lifespan and is associated with neuroanatomical adaptations thought to facilitate more accurate spatial memory (Couto et al., 2023; Farnworth et al., 2024; Young and Montgomery, 2020). Specific pollen plants are frequently visited by individuals in a spatially and temporally specific manner (Boggs et al., 1981; Estrada and Jiggins, 2002), which suggests a sophisticated capacity for spatial memory and navigation. In particular, mark-and-release studies of wild *Heliconius* show that individuals repeatedly return to the same pollen sources over successive days for prolonged periods (Ehrlich and Gilbert, 1973; Finkbeiner, 2014; Gilbert, 1975; Mallet, 1986a). In contrast, their non-pollen-feeding relatives are thought to be more vagrant foragers, although data on the foraging ecology of these genera are limited. This habit of returning faithfully to profitable food sources suggests an ability to use environmental cues that encode spatial information with heightened accuracy, and their capacity to form spatial memories has been confirmed experimentally (Dell'Aglio et al., 2024; Moura et al., 2023, 2024). *Heliconius* butterflies do not use individual local landmarks as cues to locate food sources, but most likely rely on the coupling of wide-field visual scenes and celestial cues (Moura et al., 2024).

To support this capacity for spatial learning, *Heliconius* have evolved expanded mushroom bodies (Couto et al., 2023; Montgomery et al., 2016; Sivinski, 1989), the site of learning and memory in insects (Zars, 2000). In *Heliconius*, mushroom bodies are four-fold larger in size, relative to overall brain size, and contain up to eight times more Kenyon cells (intrinsic mushroom body neurons) than early branching genera within Heliconiini (Couto et al., 2023; Young and Montgomery, 2023). This expansion is associated with sensory specialisation towards processing visual input (Couto et al., 2023), likely driven by a need to learn the spatial locations of pollen resources in visually complex, heterogeneous forest habitats (Ehrlich and Gilbert, 1973; Gilbert, 1972). Increases in the population of Kenyon cells are also predicted to facilitate the storing of more landscape representations, essential for memorising many distinct visual cues to support learned foraging routes (Ardin et al., 2016). What behavioural mechanisms are used by *Heliconius* to capture visual information used in spatial foraging is currently unclear, but the prevalence of orientation flights/walks in Hymenoptera leads to the hypothesis that *Heliconius* will also display analogous orientation behaviours to support spatial learning.

Although it is currently unknown whether *Heliconius* or other butterflies display similar behavioural strategies to the orientation behaviours of Hymenoptera, distinct exaggerations of flight behaviour in *Heliconius* have been described around nocturnal roosts (Jones, 1930; Mallet, 1986b), which, like pollen resources, are thought to be memorised and located visually (Jones, 1930). Therefore, we predicted that *Heliconius* display exaggerated flight behaviours around new floral resources, which will display some features similar to those described for orientation flights in Hymenoptera. Given the heightened importance of spatial learning in *Heliconius* compared with other Heliconiini, we also predict that these behaviours may be expressed to a greater degree in this genus. To explore these orientation-like behaviours in detail, we first described flight behaviour in *Heliconius* and non-*Heliconius* butterflies when facing a new floral resource. We performed a series of experiments designed to test some key predictions of orientation-like behaviours in both *Heliconius* and their non-pollen-feeding relatives. Specifically, we explored (i) the presence of circling, or other behaviours that involve returning to face a resource location multiple times; and (ii) changes in the expression of these behaviours with experience. Although these behaviours were observed in some degree by all species, we report that *Heliconius* species spent more time performing these behaviours and showed evidence of memorising the resource location more quickly than non-pollen-feeding Heliconiini.

## MATERIALS AND METHODS

### Butterfly rearing

We used two species of *Heliconius*, *H. erato* (Linnaeus 1758) and *H. melpomene* (Linnaeus 1758), and, for comparison purposes, two closely related species that do not have enlarged mushroom bodies and do not feed on pollen, *Dryas iulia* (Fabricius 1775) and *Dryadula phaetusa* (Linnaeus 1758) (Couto et al., 2023). Stock populations of each species were established in outdoor insectaries at the Smithsonian Tropical Research Institute, Gamboa, Panama. Adults were collected from wild populations around the institute, and eggs and caterpillars were raised on their respective hostplants (*H. erato*, *D. iulia* and *D. phaetusa* on *Passiflora biflora*, *H. melpomene* on *Passiflora menispermifolia*). Emerged adults were kept in standardised conditions (2 m^3^ outdoor cages with natural sunlight) and fed with natural flowers until the experiments.

### Experimental procedure

Experiments were performed in an experimental cage (3×3×2 m; Fig. S1) with a group of six butterflies of a single species each time. Individuals were marked and sexed to allow individual identification. The food resource available in the cage consisted of three pots of flowering plants: *Stachytarpheta mutabilis* and/or *Pentas lanceolata*, sparsely positioned in the cage (Fig. S1). These plant species are well known to attract butterflies, are regularly used to feed Heliconiini butterflies and provide both nectar and pollen (Estrada and Jiggins, 2002). The butterflies had 2 days to become habituated in the new cage, and could freely feed from the flowers.

After the cage habituation period, a new floral resource was placed in the centre of the cage each morning (∼09:00 h), when all species are motivated to feed (Dell'Aglio et al., 2024). The experimental floral resource consisted of inflorescences of *Lantana camara* freshly collected and put in a 50 ml Falcon tube with water. Two action cameras (GoPro^©^) were positioned to film approaches to the flowers from the side and from below (Fig. S1). To capture the first interactions between the butterflies and the resource, we recorded the first 40 min after this resource was introduced. Subsequently, the cameras and the experimental floral resource were removed from the cage. This procedure was repeated over three consecutive days.

Behavioural video footage was analysed using BORIS software (Behavioral Observation Research Interactive Software; Friard and Gamba, 2016). The behaviours recorded and described below (Table 1) were defined during previous observations in the wild. Using the videos recorded from the side of the floral resource, we recorded: (i) time until first feeding (time to find the new resource), (ii) duration and number of feeding attempts, and (iii) duration and number of ‘hovering’ and ‘circular flight’ behaviours around the flowers (see Table 1 for full description). To calculate the average duration for which a behaviour was expressed, we used the total duration of the behaviour (seconds) by the number of occurrences of the behaviour. To calculate the number of hovering flights per feeding attempt, we used the total number of hovering occurrences by the total number of feeding occurrences. Feeding bouts were annotated using tabular events data from BORIS in which periods of consecutive feeding occurrences (maximum time between feeding events to be included in the same bout was stipulated to be 3 min) were grouped per individual. Using the videos captured from below the floral resource, we recorded the diameter (maximum observable distance from individual take-off) and duration of the circular flights. We note that the field of view of the camera was less than the total diameter of the cage, and as a result the diameters of the largest circular flights could not be quantified, which may have impact our later analyses of how this trait changes with time.

**Table 1.** Description of behaviours recorded during experiment

Behaviour	Description	Videos
Circle flight	Flying in exaggerated circles around the flower, associated with a feeding bout.	Movie 1
Hovering	Rapid flying up and down, and/or backward and forward, with a momentarily fixed heading direction and net zero positional movement, while rotating closely to the flower.	Movie 2
Feeding bout	Multiple feeding visits to the same resource during a short period of time, intermitted with circle flights and hovering.	
Feeding	Landed on flower with proboscis expansion inside flower tube. Head movements up and down can be observed.	Movies 1 and 2

To explore the importance of floral odours to these behaviours, we added a final trial for each species with naive butterflies, repeating the same methodology, but using an artificial flower (orange foam sheet with a 1 ml Eppendorf tube for solution attached in the centre) instead of *Lantana* flowers. In this experiment, all real flowers were removed from the cage and only the new artificial flower was positioned in the centre of the cage. On the first and second days, the artificial flower contained a sugar–water solution (20% sugar and 80% water), and on the third day, we used a clean, previously unused artificial flower that was empty. During this trial, we recorded the same behaviours as listed above. Naive butterflies are not strongly attracted to artificial flowers, and typically must be trained to use them. Therefore, we expected feeding attempts from this experiment to be low in number, as we focused on innate behaviours of untrained individuals. As such, quantitative data from this experiment are limited. However, it allowed us to assess whether visual cues associated with the feeder would stimulate flight behaviours around the potential food source, or whether olfactory cues were essential to initiate them.

This work was carried out under permission from Ministerio del Ambiente, Panama (permit ARB-110-2022).

### Statistical analysis

We used generalised linear mixed models (GLMMs) implemented with the lme4 package in R (Bates et al., 2015), transforming data to a log-normal distribution to fit the normality assumption. We included day and species as fixed factors, and individuals and group (butterflies tested in the same day) as random factors. Significance of fixed factors was determined through an analysis of deviance test (Type II Wald χ^2^ test), followed by Tukey's *post hoc* tests in R (https://www.r-project.org/). Count data were analysed using a generalised linear model (GLM) with a Poisson family followed by analysis of deviance tests. To test for differences between pollen-feeding and non-pollen-feeding Heliconiini, some analyses were calculated grouping the species in ‘*Heliconius*’ (*H. erato* and *H. melpomene*) and ‘non-*Heliconius*’ (*D. iulia* and *D. phaetusa*). Twenty-eight individuals that did not interact with the flowers were removed from the dataset. Therefore, results are based on a final dataset of 14 *H. erato* (8 females, 6 males), 19 *H. melpomene* (6 females, 13 males), 15 *D. iulia* (6 females, 9 males) and 15 *D. phaetusa* (5 females, 10 males), all of which interacted with the flowers on all 3 days of experiment (total of 63 out of 91 individuals)*.* None of the behaviours observed were influenced by sex (all *P*>0.05), so this factor was removed from the analysis.

## RESULTS

### Heliconiini display exaggerated flight behaviours around floral resources

**Fig. 1.**
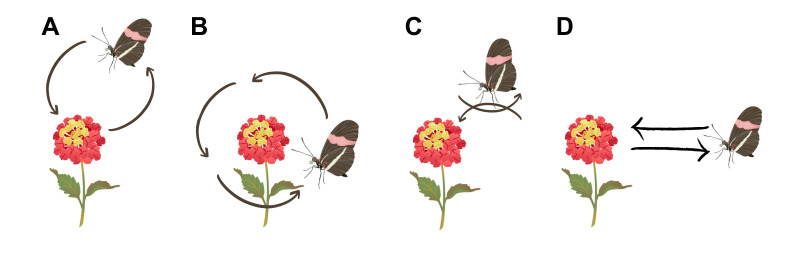
**Behaviours of *Heliconius erato* observed towards a newly introduced floral resource, *Lantana camara*.** (A) Circle flight moving away from the flower and (B) circle flight moving around the flower, both varying up to 2 m in diameter, but were constrained by the limits of the cage. (C) Hovering, occurring over 1 to 3 cm from the flower. (D) Feeding bouts. *Heliconius erato* drawing by Amaia Alcalde Anton.

We observed three prominent patterns of flight behaviour prior to, during and after feeding, which we refer to as ‘circle flights’, ‘hovering’ and ‘repetitive feeding bouts’ (Fig. 1, Table 1; Movies 1 and 2). During circle flights, butterflies moved in large circles radiating around the newly introduced floral resource, before an individual returned to the original flower resource they previously fed from (Fig. 1A,B; Movie 1). These exaggerated flights were relatively common across individuals (*H. erato*=7 individuals out of 14, *H. melpomene*=8/19, *D. iulia*=7/15 and *D. phaetusa*=5/14), always occurred only after the first feeding attempt, and involved either clockwise and/or anticlockwise flight directions, but always ended with individuals returning to feed after few seconds (see details below). Most individuals (all but two *D. phaetusa* and three *D. iulia*) also displayed hovering flights, typically before and after landing on the flower to feed, in which the individual had a fixed heading direction to the new floral resource but flew rapidly up and down, and/or backward and forward, with net zero movement with respect to distance from the flower (Fig. 1C; Movie 2). This hovering flight often included continuous repetition while the butterfly rotated around the flower, in an orbital pattern always facing the flower, such that an individual experienced a wide range of viewing angles around the flower (Movie 2). This occurred before most initial feeding attempts, and also following most circle flights between consecutive feeding attempts. Together with periods of stationary feeding, these behaviours occurred within notable feeding bouts, characterised by several feeding visits to the same resource during a certain time (performed by *H. erato*=7 individuals out of 14, *H. melpomene*=13/19, *D. iulia*=12/15 and *D. phaetusa*=10/14; Fig. 1D). During these bouts, individuals would fly away from the flowers but return multiple times to feed, typically performing repeated circle flights and hovering between consecutive feeding periods.

### *Heliconius* learn to utilise a new floral resource faster than outgroups

**Fig. 2.**
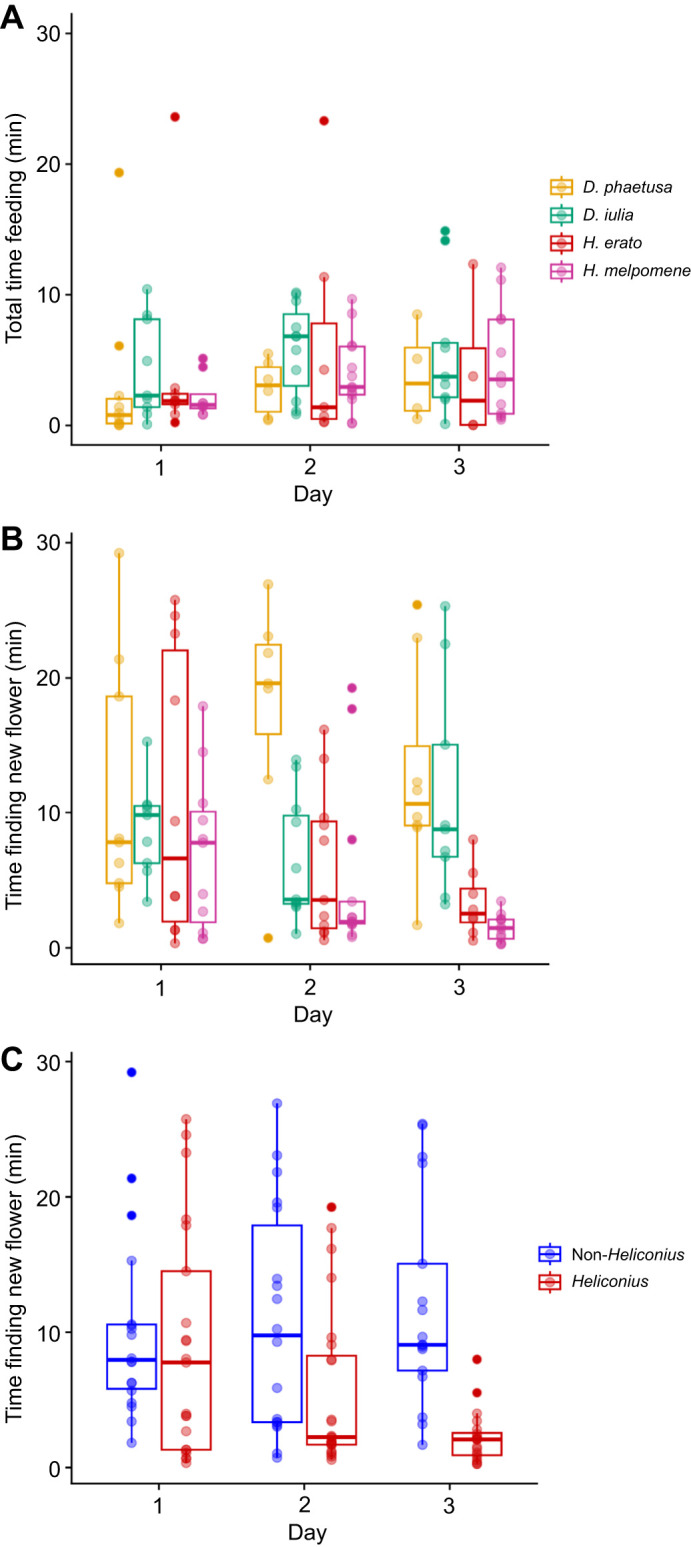
***Heliconius* are faster in locating the floral source.** (A) Total time in minutes spent in feeding on the new flower for each species per day. (B,C) Time in minutes to find the new flower per day by (B) species and (C) Heliconiini group (‘*Heliconius*’: *H. erato* and *H. melpomene*; ‘non-*Heliconius*’: *Dryas iulia* and *Dryadula phaetusa*). Each dot is an individual. Darker dots indicate that individuals overlap. Box plots show median, upper and lower quartiles, maximum and minimum.

All species spent similar times feeding on the newly introduced floral resource across all days (species, χ^2^_3_=5.7, *P*=0.125; day, χ^2^_2_=2.4, *P*=0.300; species×day, χ^2^_6_=6.4, *P*=0.374; Fig. 2A). They also performed a similar number of feeding events (species: χ^2^_3_=6.3, *P*=0.096, day: χ^2^_2_=2.7, *P*=0.255; species×day, χ^2^_6_=2.9, *P*=0.815), suggesting similar motivation and exposure to the floral cues and/or reward. However, species differed in the time taken to discover the new flower resources once introduced to the cage (species, χ^2^_3_=31.03, *P*<0.001; day, χ^2^_2_=5.48, *P*=0.064; species×day, χ^2^_6_=11.65, *P*=0.070; Fig. 2B). On the first day that the floral resource was introduced, all four species had the same average time to find the new flowers (species, χ^2^_3_=0.98, *P*=0.805), suggesting that sensory perception of the resource did not vary between species. In contrast, on the second day, some species returned to the new resource quicker than others (species, χ^2^_3_=9.76, *P*=0.021; *post hoc*: *D. phaetusa* – *H. melpomene*, *P*=0.054), and by the third day, both *H. erato* and *H. melpomene* were faster in locating and feeding the resource than both *D. iulia* and *D. phaetusa* (species, χ^2^_3_=43.94, *P*<0.001, *post hoc D. phaetusa* and *D. iulia* versus *H. erato* and *H. melpomene*, *P*<0.05). Grouping *Heliconius* and non-*Heliconius* species (group, χ^2^_1_=30.58, *P*<0.001; day, χ^2^_2_=5.60, *P*=0.060; group×day, χ^2^_2_=8.54, *P*=0.013; Fig. 2C) confirmed a significant difference between pollen-feeding and non-pollen-feeding groups on the third day (*post hoc*: day 3, *P*<0.001).

### Heliconiini display repeated visits to the same flower

**Fig. 3.**
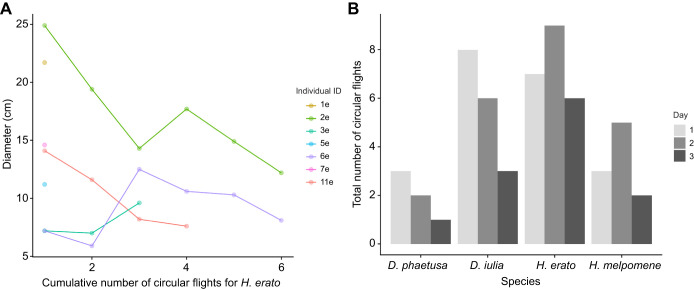
**All species performed circular flights.** (A) Diameter of the circular flight performed by *Heliconius erato* during the experiment. Each colour is an individual. (B) Total number of circular flights per species per day.

The mean±s.e.m. duration of feeding bouts for *Heliconius* butterflies was 8.44±0.99 min, with an average of 6.15±0.57 visits per bout. For non-*Heliconius* butterflies, the mean duration of feeding bouts was 7.06±0.86 min, with an average of 8.67±1.08 visits per bout. There was no difference between the groups for feeding bout duration (χ^2^_1_=0.52, *P*=0.469) or for the number of feeding events during bouts (χ^2^_1_=3.63, *P*=0.056). During intervals between feedings, individuals would fly around the cage, performing circle flights and/or hovering. Although when analysing *H. erato* separately, some individuals showed a decrease in diameter of the circle with each flight (χ^2^_1_=7.51, *P*=0.006; Fig. 3A), within the constraints of the camera field of view and cage size, the diameter of circular flights was not different across days between species (species: χ^2^_3_=5.96, *P*=0.113, day: χ^2^_2_=1.58, *P*=0.453; species×day: χ^2^_6_=5.85, *P*=0.439) or between grouped *Heliconius* and non-*Heliconius* species (group: χ^2^_1_=0.02, *P*=0.883, day: χ^2^_2_=1.55, *P*=0.458; group×day: χ^2^_2_=2.91, *P*=0.233). Species varied in the number of circular flights performed while exploring the new floral resource (species: χ^2^_3_=11.57, *P=*0.008, day: χ^2^_2_=3.55, *P*=0.169; Fig. 3B), with *H. erato* performing more circular flights than *D. phaetusa* (*post hoc*: *P*=0.024). However, when grouped, there was no difference between *Heliconius* and the outgroup Heliconiini (group: χ^2^_1_=1.47, *P*=0.223, day: χ^2^_2_=3.55, *P*=0.169).

### Increase in hovering flights in *Heliconius* relative to closely related non-*Heliconius* species

**Fig. 4.**
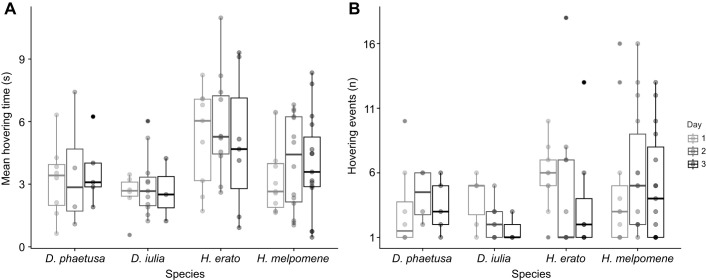
***Heliconius* butterflies performed more hovering behaviour.** (A) Mean hovering time in seconds in front of the new flower for each species per day. (B) Total number of hovering events per individual for each species per day. Each dot is an individual, darker dots indicate that individuals overlap. Box plots show median, upper and lower quartiles, maximum and minimum.

Although circle flight patterns were relatively consistent across species, *H. erato* and *H. melpomene* spent substantially more time hovering in front of the new floral resource overall (species: χ^2^_3_=10.33, *P*=0.015; day: χ^2^_2_=1.06, *P*=0.588; species×day: χ^2^_6_=1.2, *P*=0.976; Fig. 4A; Heliconiini group: χ^2^_1_=4.57, *P*=0.032; day: χ^2^_2_=1.07, *P*=0.583; group×day: χ^2^_2_=0.94, *P*=0.623; Fig. S2). Moreover, *Heliconius* also performed more hovering events than both outgroup Heliconiini (species: χ^2^_3_=27.5, *P*<0.001; day: χ^2^_2_=2.23, *P*=0.327; *post hoc*: *D. phaetusa* versus *H. melpomene*: *P=*0.019, *D. iulia* versus *H. melpomene*: *P*<0.001 and *D. iulia* versus *H. erato*: *P*=0.003, Fig. 4B), and this difference was robust to grouping *Heliconius* and non-*Heliconius* species (group: χ^2^_1_=23.86, *P*<0.001; day: χ^2^_2_=1.65, *P*=0.438). Both *Heliconius* species also showed a higher amount of hovering per feeding attempt than both non-*Heliconius* species when grouped (group: χ^2^_1_=24.54, *P*<0.001; day: χ^2^_2_=8.93, *P*=0.011; group×day: χ^2^_2_=6.30, *P*=0.042; Fig. S3A), and also when species were compared without grouping (species: χ^2^_3_=62.61, *P*<0.001; day: χ^2^_2_=6.18, *P*=0.045; species×day: χ^2^_6_=9.09, *P*=0.168; Fig. S3B). A particularly strong effect was found between *Heliconius* and *Dryas* (*post hoc*: all *P*<0.001). Artificial feeders provoked limited numbers of feeding attempts among all species, which was expected as all individuals were naive with regards to feeder use. However, hovering flights were still observed in the absence of floral cues, and in the absence of food rewards, for all four species when approaching the artificial feeders, suggesting that floral odours are not essential to trigger this behaviour (Fig. S4, Movie 3).

## DISCUSSION

The independent evolution of spatially and temporally faithful foraging behaviours in *Heliconius*, in comparison with rich data from Hymenoptera, provides a unique opportunity to understand links between the evolution of spatial memory and the behavioural mechanisms used to capture environmental information. We predicted that Heliconiini butterflies – in particular, the genus *Heliconius* – must have evolved behavioural traits to support capture of visual landscape cues. In that context, we described a series of exaggerated flight behaviours that are expressed when foraging on a newly discovered resource. We interpret these behaviours as being similar to orientation flights observed in Hymenoptera, and discuss this argument below, alongside alternative interpretations and experimental limitations.

As observed during orientation behaviours in Hymenoptera, circle flights are a major structural component of learning flights and it is likely that position cues are learnt during these repetitive flights (Collett et al., 2013; Zeil et al., 1996). Similar behaviour was observed in Heliconiini butterflies, which also repeatedly leave and turn back to the food source, flying in circles and in different directions (clockwise/counterclockwise). In bees, they gradually fly faster and their flight loops expand until they cover many metres, and this expansion may facilitate learning larger, more distant visual features, which can be used for navigation to and from the nest (Capaldi et al., 2000; Menzel et al., 2006). Although performing similar wild experiments in butterflies is not feasible, the behaviours we describe in our experiments could be analogous to the circular flights of bees and might enable Heliconiini butterflies to learn visual cues of the surroundings. However, we note that the limited field of view imposed by our caged experimental design restricted our quantification of the details (size and number) of the flight loops. Moreover, our experimental period of 3 days is limited compared with the temporal consistency of *Heliconius* foraging routes in the wild, which can extend for many weeks or months (Ehrlich and Gilbert, 1973; Finkbeiner, 2014; Gilbert, 1975; Mallet, 1986b). As such, it remains possible that over longer time periods, more pronounced temporal patterns may become apparent.

The circular flights we observed were only displayed after an individual had initially fed, and occurred in between repeated feeding from the same flower over a short time frame. These repetitious floral visits are referred to as foraging bouts, and involve an individual repeatedly leaving and returning to the same resource (Dell'Aglio et al., 2024). Previously, *Heliconius* have been found to have longer feeding bouts with more repeated visits (Dell'Aglio et al., 2024). Here, this effect was not found, but this is likely explained by the duration of feeding bouts in this experiment being shorter than previous work owing to the reduced observation time (40 min in the present study versus 8 h in Dell'Aglio et al., 2024). Regardless, by repeatedly revisiting the same resource over a short time frame, while performing multiple circular flights, an individual increases the capture of visual information around a positively rewarding resource, potentially aiding formation of spatial memories in an manner analogous to the orientation flights of bees and wasps (Zeil et al., 1996). Experiments in *Drosophila*, the most tractable model system for insect neurobiology, have indeed shown that forming long-term memory requires repetitive experience spread over time (Jacob and Waddell, 2020). Therefore, the necessity to accumulate sensory information around rewarding resources, and to subsequently reduce searching behaviour for new flowers, particularly for pollen-feeding *Heliconius*, may promote this repetitious feeding behaviour. In the wild, this is likely further supported by the reliability of pollen provision from the preferred resources of *Heliconius*, which also benefits the plants themselves by promoting flower constancy to ensure correct pollen transfer (Papaj and Lewis, 1993).

An additional feature of the Heliconiini flight behaviours we observed, which has a less clear analogy to hymenopteran behaviours, was the hovering flight. All Helconiini performed these rapid movements, before, during and after feeding bouts. However, we found evidence that *Heliconius* butterflies spent longer periods hovering around the newly introduced flower in general, compared with non-pollen-feeding relatives, suggesting a possible extension of ancestral floral inspection and landscape information capture behaviours. During hovering, the butterflies also rotate around the flower, providing a wide range of views around the resource (Movie 2). To our knowledge, this kind of hovering behaviour around floral resources has not been described previously in Lepidoptera outside species with more distinct hovering flight patterns. For example, comparing hovering hawkmoths and perching bumblebees, it was noted that hovering might be a time-minimising strategy during foraging, as hawkmoths were faster in collecting nectar from a higher number of flowers compared with bumblebees (Dreisig, 1997). However, in this case, hawkmoths are able to feed while hovering, by extending elongated proboscises, whereas in Heliconiini, hovering is distinct from successful feeding. According to models of flight kinematics and aerodynamic power, hovering is more costly than flying (Casey, 1981; Ellington, 1984; Willmott and Ellington, 1997), suggesting that this behaviour likely incurs significant costs to the individual. If true, then the expectation is that there must be some benefit to the individual performing this behaviour.

Hovering in butterflies is observed not only when searching for food sites, but also during mate selection and oviposition (Betts and Wootton, 1988; Dudley, 1990; Nentwig, 1993). Hovering in *Heliconius* is well documented as being an important mating behaviour for female acceptance, as during courtship males hover over perched females (Klein and de Araújo, 2010; Kuo et al., 2024; Southcott and Kronforst, 2018). However, its function during mating is likely to be different from its function during foraging. During hovering in mating, males slightly splay their wings, separating the forewing from the hindwing to expose their androconia, which has been interpreted as enabling greater release and distribution of mating pheromones (Darragh et al., 2017; Klein and de Araújo, 2010). During hovering described here, before landing on the flower, this splayed wing movement was not observed (Movie 2). Hovering during foraging is also observed in both males and females, whereas females do not hover during mating. *Heliconius* also show a hovering-like behaviour (described as fanning) when they approach a roost (Mallet, 1986b). In this context, they hover when approaching other individuals of the roost very closely, with probable antennal and wing contact, and in contrast, these individuals do not present any reaction to it. Mallet (1986b) suggested that in this case hovering could be a modified courtship behaviour used in recognition of conspecifics, as some, but not all, species of *Heliconius* roost together. However, not all *Heliconius* aggregate in groups at night, but all *Heliconius* do return to one of a small number of roost sites each night (Finkbeiner, 2014), which in itself requires spatial memory. Here, we hypothesise that *Heliconius* may use hovering around both roost sites and plant resources, to gather visual information about these locations for spatial memory, as suggested in early accounts of roosting behaviour (Jones, 1930).

Although all four species of Heliconiini butterflies we tested demonstrate hovering, the *Heliconius* spp., which tended to spend more time hovering in general, also showed evidence of more rapid learning by significantly decreasing the time taken to discover the new floral source over consecutive days. In previous learning studies, *Heliconius* have been demonstrated to readily associate shapes and colours with food rewards (Dell'Aglio et al., 2016, 2022; Swihart and Swihart, 1970; Toure et al., 2020; Young et al., 2024). In cage experiments, *Heliconius* also exhibit good spatial learning, memorising the location of food rewards at different spatial scales (Moura et al., 2023, 2024). This is consistent with the importance of spatial learning for pollen feeding, and evidence from wild studies that they are able to find high reward pollen resources, return to a limited number of roosts at night, and maintain stable and limited home ranges of 100 m^2^ to 1 km^2^ (Mallet, 1986b; Murawski and Gilbert, 1986) through strong site fidelity and homing after displacement (Moura et al., 2022).

Although outgroup Heliconiini have less pronounced specialisations for spatial foraging, it is highly likely they have some capacity to learn spatial cues. These species also forage for resources, and occupy the same habitats and overlapping hostplants and nectar sources as *Heliconius* (Gilbert, 1975), while some are also territorial (Benson et al., 1989). As such, it is likely that they also have a degree of spatial memory, but *Heliconius* have extended and refined this capacity in specific ways. Indeed, in comparative learning and memory experiments, *Heliconius* outperform other Heliconiini only in very specific contexts, such as non-elemental learning and long-term visual memory (Couto et al., 2023; Hodge et al., 2025; Young et al., 2024), whereas in most other contexts they perform similarly (Hodge et al., 2025; Toure et al., 2020; Young and Montgomery, 2023; Young et al., 2024). Therefore, our Heliconiini data may suggest that similar flight behaviours are more phylogenetically widespread than currently appreciated. It is thus perhaps not surprising that *Heliconius* differ from other Heliconiini by exaggeration of common flight behaviours, rather than through innovation of new ones. Although alternative hypotheses for these elongated periods of hovering in *Heliconius* could be proposed, such as the gathering of information about quality and quantity of nectar, or inspecting flowers for predators such as crab spiders, which are common in *Lantana camara* (Dukas and Morse, 2003; Nentwig, 1993), these would not explain the difference in hovering we observed across species, or indeed the persistence of circular flight patterns associated with feeding during feeding bouts. Nevertheless, these alternative hypotheses merit consideration and future experimentation.

In summary, our findings add to existing evidence that demonstrates specialisations in Heliconiini, and *Heliconius*, foraging behaviours. Based on predictions derived from the behavioural adaptations in Hymenoptera with known specialisations in spatial memory, we have described orientation-like behaviours in Heliconiini butterflies. We suggest that these behaviours are important for the capture and recall of visual landscape cues, but additional experiments are needed to provide data that directly support this function in spatial learning and memory. There are challenges of working on spatial foraging outside of Hymenoptera, which have the convenience of allocentric foraging around nests and highly reliable foraging behaviours. The same is not true in foraging insects such as butterflies, which require caged experimental design. Establishing methods of tracking individuals over time and space in the wild such as the use of radar (Ovaskainen et al., 2008), or manipulating wide-field landscape cues in large experimental contexts, are necessary to further pinpoint the function of these behaviours. If confirmed, however, their function in spatial foraging would add to examples of convergence in cognitive abilities (Young and Montgomery, 2020) and neuroanatomies (Couto et al., 2023; Farnworth et al., 2024) between *Heliconius* and Hymenoptera, which are both specialised to exploit stable spatial information to increase foraging efficiency (Ardin et al., 2016; Kiya and Kubo, 2011; Stieb et al., 2010). The present study on orientation-like flight behaviour in butterflies is therefore a necessary first step to understanding the behavioural mechanisms that allow *Heliconius* to learn, memorise and use spatial information of floral resources around their home range. Results from this research program will allow refined models of butterfly navigation and cognitive neuroethology.

## Supplementary Material

10.1242/jexbio.250975_sup1 Supplementary information
