# Supplementary material for: Heliconiini butterflies display flight behaviours reminiscent of orientation flights when using new floral sources
Source: J Exp Biol. 2025 Nov 5;228(21):jeb250975. doi: 10.1242/jeb.250975 (PMC12633734; doi:10.1242/jeb.250975)
Supplement: Supplementary information [file jexbio-228-250975-s1.pdf]

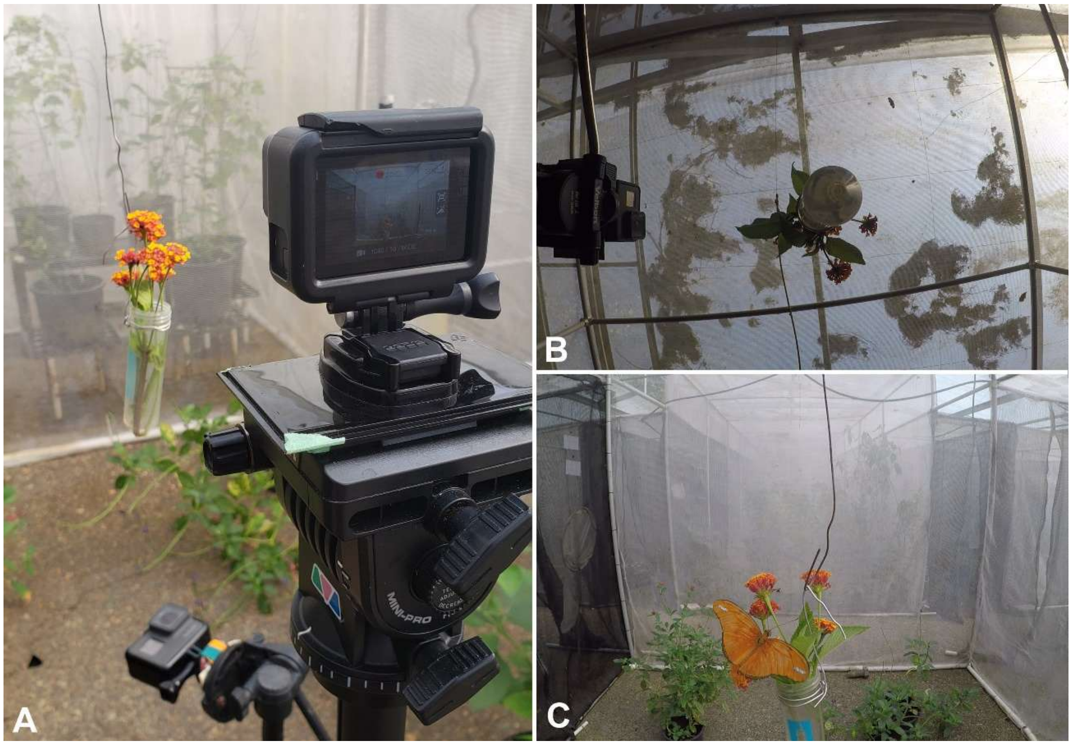

Fig. S1. (A) Experimental cage with flowers and two action cameras positioned on the side and below the experimental flower. (B) Video screenshot of the below camera. The experimental flower is at the centre. (C) Video screenshot of the side camera, showing *Dryas iulia* feeding on the experimental flower *Lantana camara*.

Journal of Experimental Biology • Supplementary information

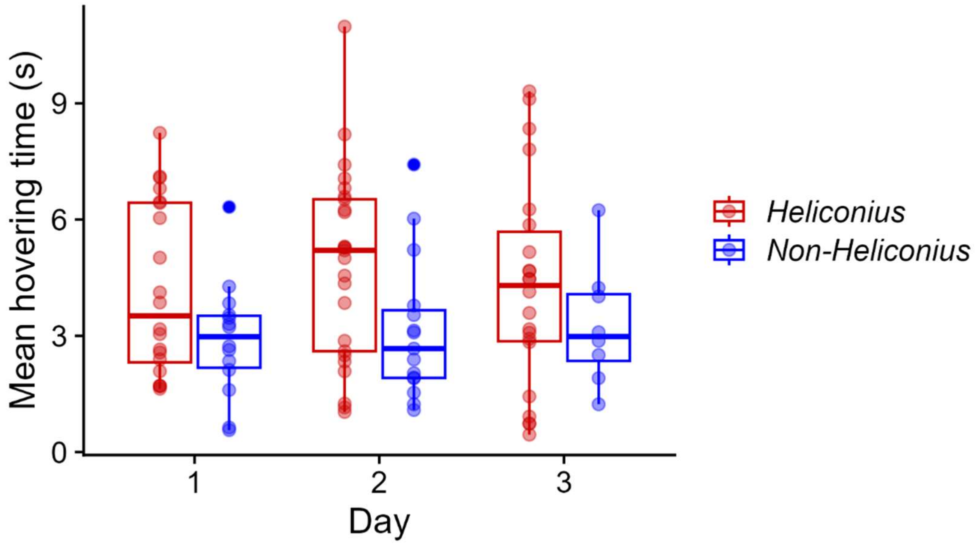

Fig. S2. Time hovering in front of the new flower for each group per day. *Heliconius* in red (*H. erato* and *H. melpomene*) and "Non-*Heliconius*" in blue (*D. iulia* and *D. phaetusa*). Each dot is an individual. Box plots show median, upper and lower quartiles, maximum and minimum.

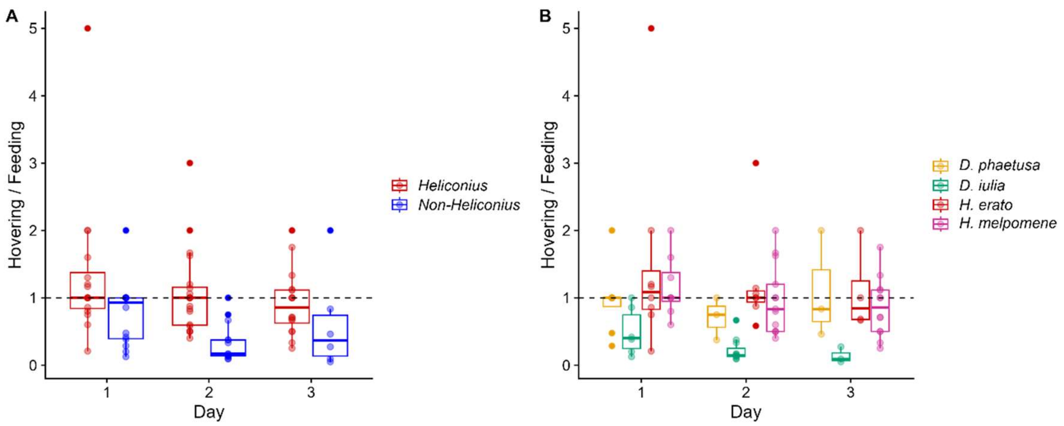

Fig. S3. (A) Number of hovering per feedings events for each Heliconiini group per day. *Heliconius* in red (*H. erato* and *H. melpomene*) and "Non-*Heliconius*" in blue (*D. iulia* and *D. phaetusa*). (B) Number of hovering per feedings events for each species per day. Each dot is an individual. Box plots show median, upper and lower quartiles, maximum and minimum.

Journal of Experimental Biology • Supplementary information

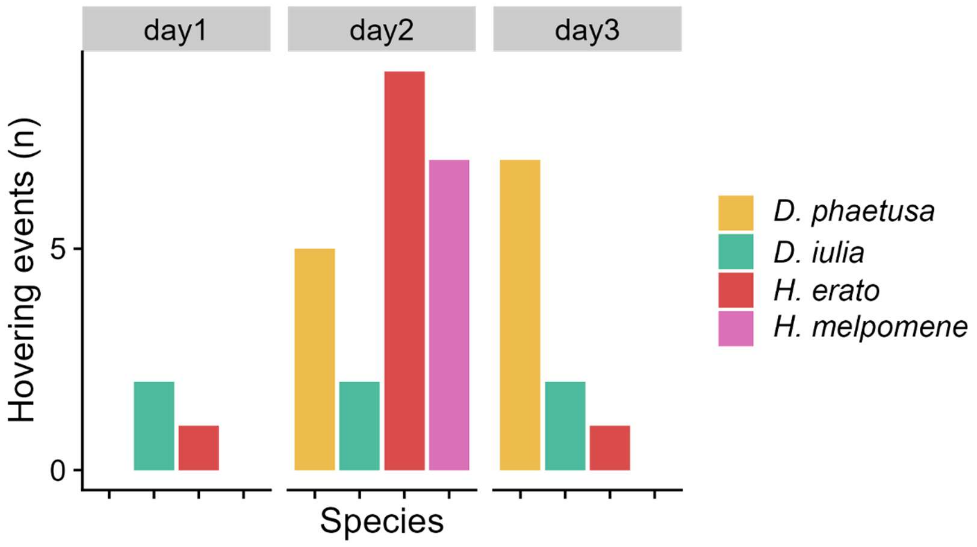

Fig. S4. Total number of hovering events towards an artificial flower for each species per day. Day 1 and 2: artificial flower contained sugar-water solution. Day 3: artificial flower was empty.

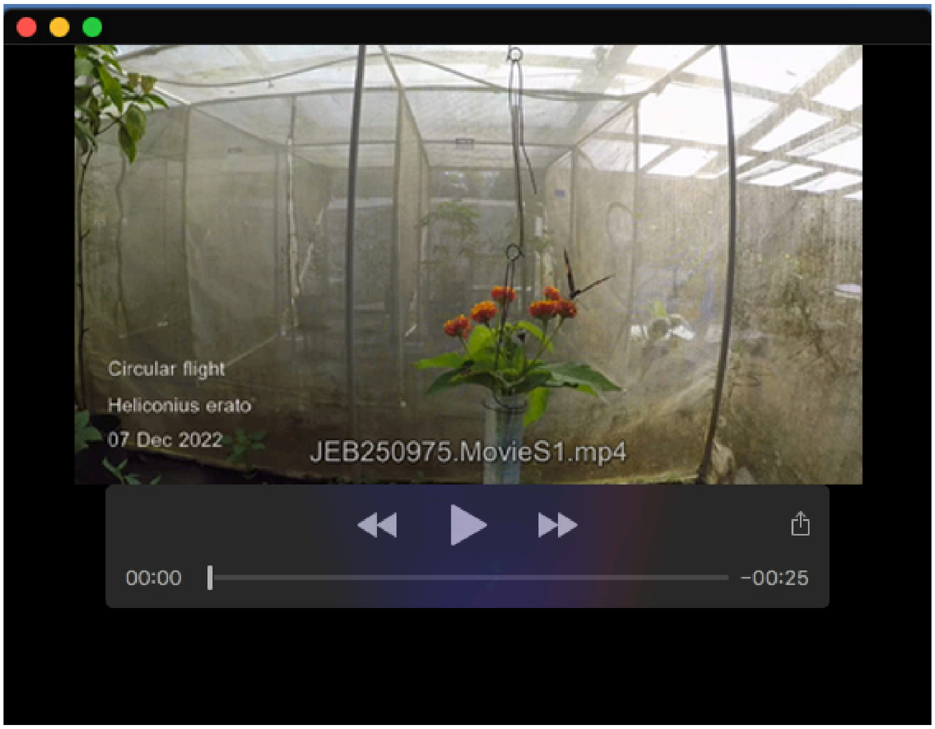

Movie 1. Example of circular flights observed during the experiments with natural flowers for the four species studied.

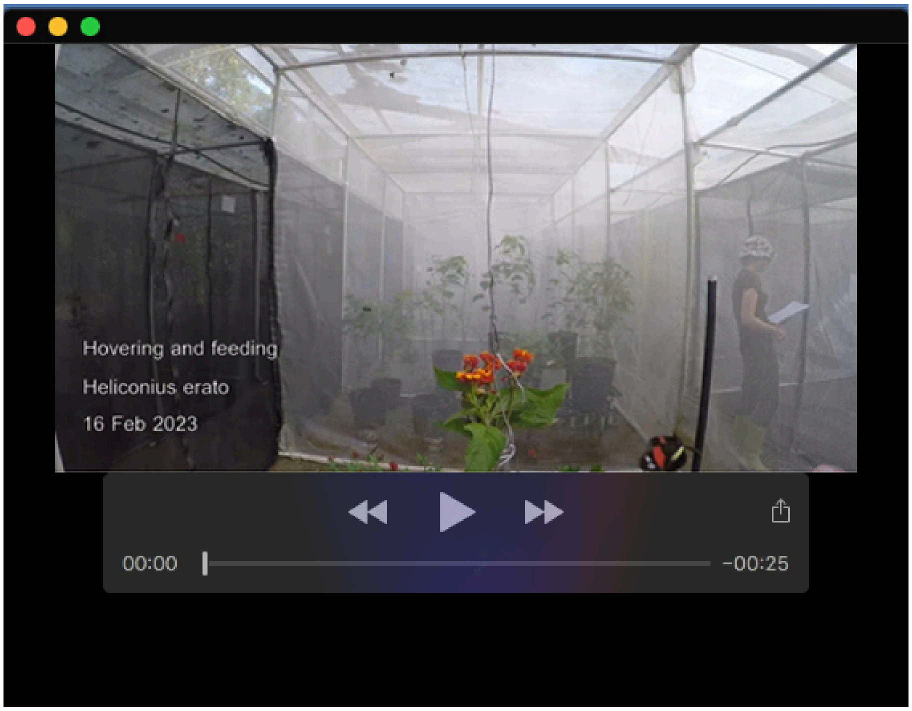

Movie 2. Example of hovering flights observed during the experiments with natural flowers for the four species studied.

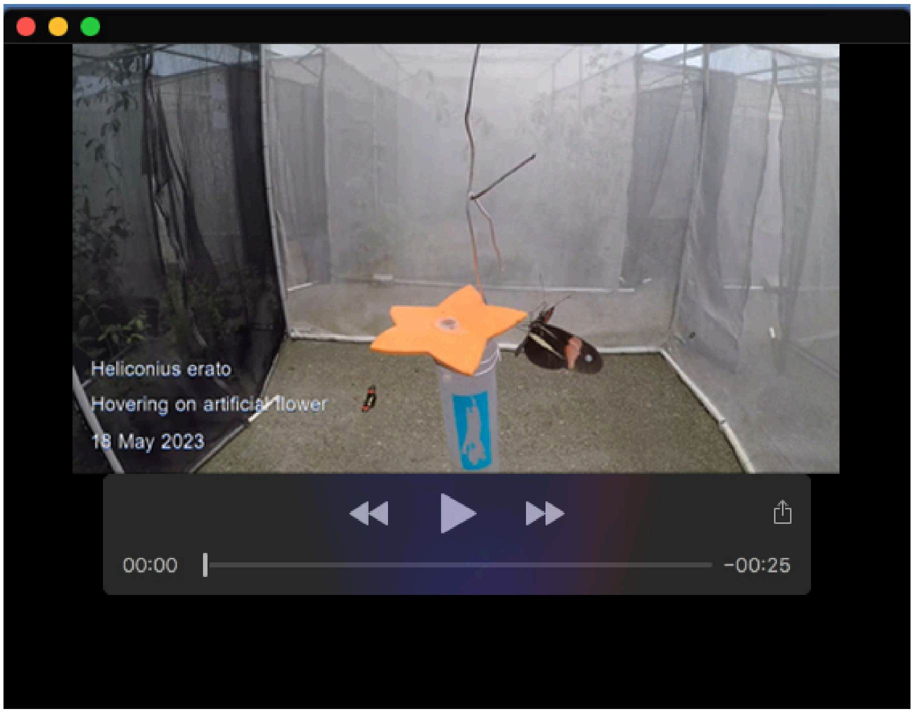

Movie 3. Example of hovering flights observed during the experiments with an artificial flower for the four species studied.
